# Genome-Wide Analysis of the bZIP Transcription Factors in Cucumber

**DOI:** 10.1371/journal.pone.0096014

**Published:** 2014-04-23

**Authors:** Mehmet Cengiz Baloglu, Vahap Eldem, Mortaza Hajyzadeh, Turgay Unver

**Affiliations:** 1 Kastamonu University, Faculty of Engineering and Architecture, Department of Genetics and Bioengineering, Kastamonu, Turkey; 2 Istanbul University, Faculty of Science, Department of Biology, Istanbul, Turkey; 3 Cankırı Karatekin University, Faculty of Science, Department of Biology, Cankiri, Turkey; East Carolina University, United States of America

## Abstract

bZIP proteins are one of the largest transcriptional regulators playing crucial roles in plant development, physiological processes, and biotic/abiotic stress responses. Despite the availability of recently published draft genome sequence of *Cucumis sativus*, no comprehensive investigation of these family members has been presented for cucumber. We have identified 64 bZIP transcription factor-encoding genes in the cucumber genome. Based on structural features of their encoded proteins, *CsbZIP* genes could be classified into 6 groups. Cucumber *bZIP* genes were expanded mainly by segmental duplication rather than tandem duplication. Although segmental duplication rate of the *CsbZIP* genes was lower than that of Arabidopsis, rice and sorghum, it was observed as a common expansion mechanism. Some orthologous relationships and chromosomal rearrangements were observed according to comparative mapping analysis with other species. Genome-wide expression analysis of *bZIP* genes indicated that 64 *CsbZIP* genes were differentially expressed in at least one of the ten sampled tissues. A total of 4 *CsbZIP* genes displayed higher expression values in leaf, flowers and root tissues. The *in silico* micro-RNA (miRNA) and target transcript analyses identified that a total of 21 *CsbZIP* genes were targeted by 38 plant miRNAs. *CsbZIP20* and *CsbZIP22* are the most targeted by miR165 and miR166 family members, respectively. We also analyzed the expression of ten *CsbZIP* genes in the root and leaf tissues of drought-stressed cucumber using quantitative RT-PCR. All of the selected *CsbZIP* genes were measured as increased in root tissue at 24th h upon PEG treatment. Contrarily, the down-regulation was observed in leaf tissues of all analyzed *CsbZIP* genes. *CsbZIP12* and *CsbZIP44* genes showed gradual induction of expression in root tissues during time points. This genome-wide identification and expression profiling provides new opportunities for cloning and functional analyses, which may be used in further studies for improving stress tolerance in plants.

## Introduction

Cucumber (*Cucumis sativus* L.), a major vegetable crop consumed worldwide, belongs to Cucurbitaceae family commonly known as cucurbits. Agricultural production of cucurbits utilizes nine million hectares of land, and yields 192 million tons of vegetables, fruits, and seeds annually (http://faostat.fao.org). Cucurbit family is a model system for the study of sex determination and plant vascular biology in which long-distance signaling events are examined using xylem and phloem sap [1]–[2]. In 2009, cucumber became the seventh plant to have its genome sequence published, following the well-studied model plant *Arabidopsis thaliana*, the poplar tree, grapevine, papaya, and the crops rice and sorghum. In 2013, a variation map of the cucumber genome at single-base resolution was generated by performing deep re-sequencing of all 115 lines with the wild cucumber genome, which was compared to the genome of cultivated cucumber [3]. These genomic resources provide new insights for understanding the genetic basis of domestication and diversity of this important crop. As a result, the released genome sequence of the cucumber encouraged the scientific research community for further study related with its structural and functional genomics, which has resulted in crop improvement and ensuring food security [4]. Consequently, the substantial findings in the aspects of both structural [5]–[9], and functional genomics [10]–[15] were reported in the vegetable model crop, cucumber.

Transcription factors (TFs) consist of sequence-specific DNA-binding domain for binding to the promoter and/or enhancer regions of corresponding genes, thereby inducing or repressing transcription of downstream target genes. TFs can be grouped into 40–60 families based on their primary and/or three-dimensional structure similarities in the DNA-binding and multimerization domains [16]–[18]. Among them, the basic leucine zipper (bZIP) transcription factor family is one of the largest and most diverse families. bZIP transcription factors have conserved bZIP domain which is composed of two structural features; a basic region that binds DNA and a leucine zipper dimerization motif [14]. The basic region of 16 amino acid residues with an invariant N-x7-R/K motif is highly conserved and responsible for nuclear localization and DNA binding. The leucine zipper is a less-conserved dimerization motif and composed of a heptad repeat of leucines or other bulky hydrophobic amino acids positioned exactly nine amino acids towards the C-terminus. Plant bZIP proteins preferentially bind to DNA sequences with an ACGT core. Currently, the bZIP family members have been identified or predicted in multiple eukaryotic genomes including plants, animals and yeasts [10], [13], [18]–[20].

It has been reported that bZIP TFs are involved in developmental and physiological processes as well as biotic/abiotic stress responses under normal and stressed growth conditions. So, they are important for various plants to withstand adverse environmental conditions [14], [21]. As developmental processes, bZIP TFs play crucial roles in organ and tissue differentiation [22]–[26], cell elongation [27]–[28], nitrogen/carbon and energy metabolism [29]–[31], unfolded protein response [32]–[33], seed storage protein gene regulation [34] somatic embryogenesis [35]. On the other hand, bZIP TFs have also been regarded as important regulators in response to various abiotic stresses such as drought, high salinity and cold stresses in Arabidopsis [36]–[41], rice [42]–[50], wheat [51], tomato [52]–[53], soybean [54]–[56], pepper [57], bean [58], barley [59] and maize [60]. However, little is known about the genome-wide survey and expression patterns of this gene family in cucumber. The genome-wide survey and identification studies from Arabidopsis [10], castor bean [11], maize [12], rice [13], sorghum [14], algae, mosses, ferns, gymnosperms and angiosperms [15] are a few examples for bZIP TFs.

On the other hand, a draft of the *Cucumis sativus* L. genome sequence was reported recently [1]. Cucumber has seven pairs of chromosomes and a haploid genome of 367 Mbp, which is smaller than other species in Cucurbitaceae family. Its small genome makes itself as a model for functional genomics of vegetable crop. However, to our knowledge, no *bZIP* gene has been identified and isolated in cucumber so far. In addition, only limited data are available on genome-wide identification and their characterizations in the cucumber genome. Therefore, the genome-wide identification and expression analysis of the cucumber *bZIP* gene family in cucumber is one of the important issues to study. Here, we identified the bZIP family members in cucumber based on the complete genome sequence analysis. Then, we have identified the genomic distribution and conserved motifs of *bZIP* gene family. Consequently, we analyzed the expression patterns of these family members by using the publicly available expression and experimental data. This extended analysis is the first comprehensive study of the *bZIP* gene family in cucumber and provides valuable information for further exploration into the functions of this significant gene family in cucumber. In addition, these results provide information about the relationship between evolution and functional divergence in the bZIP family.

## Materials and Methods

### Sequence Retrieval and Identification of bZIP-domain Proteins from Cucumber

Three different approaches were applied to identify putative bZIP proteins from *C. sativus* L. Initially, 741 amino acid sequences encoding bZIP transcription factors from seven plants (*A. thaliana, Carica papaya, Oryza sativa subsp. japonica, Populus trichocarpa, Sorghum bicolor, Vitis vinifera* and *Zea mays*) were retrieved from plant transcription factor database 3.0 (plntfdb.bio.uni-potsdam.de) [61]. These sequences were used to identify homologous peptides from cucumber by performing a BLASTP search at PHYTOZOME v9.1 database (www. phytozome.net) using default parameters, [62]. In addition, the database was searched using the keywords ‘bZIP’. Moreover, The Hidden Markov Model (HMM) profiles of the bZIP domains in the Pfam database (http://pfam.sanger.ac.uk) were searched against the PHYTOZOME database of *C. sativus*. Similarity searches were also performed through TBLASTN at NCBI database against the EST sequences of *C. sativus* genome to eliminate possible exclusions of any additional bZIP members. All hits with expected values less than 1.0 were retrieved and redundant sequences were removed using the decrease redundancy tool (web.expasy.org/decrease_redundancy). Each non-redundant sequence was checked for the presence of the conserved bZIP domain by SMART (http://smart.emblheidelberg.de) [63] and Pfam (http://pfam.sanger.ac.uk) searches.

### Chromosomal Location, Gene-structure Prediction, and Estimation of the Genomic Distribution

Specific chromosomal positions for the genes encoding these CsbZIP proteins were determined by BLASTP search of the *C. sativus* sequences against the PHYTOZOME database by using default settings. The genes were plotted separately onto the seven cucumber chromosomes according to their ascending order of physical position (bp), from the short-arm telomere to the long-arm telomere and finally displayed using MapChart [64]. Segmental duplications were identified based on the method of Plant Genome Duplication Database [65]. Briefly, BLASTP search was executed against all predicted peptide sequences of *C. sativus* and top 5 matches with ≤1e-05 was identified as potential anchors. Collinear blocks were evaluated by MCScan, and alignments with ≤1e-10 were considered as significant matches [65]–[66]. Tandem duplications were characterized as adjacent genes of same sub-family located within 10 predicted genes apart or within 30 kbp of each other [66]–[67]. The exon-intron organizations of the genes were determined using Gene-Structure Display Server (gsds.cbi.pku.edu.cn) [68] through comparison of their full-length cDNA or predicted coding sequence (CDS) with their corresponding genomic sequence.

### Sequence Alignment, Phylogenetic Analysis and Identification of the Conserved Motifs

The amino acid sequences were imported into MEGA5 [69] and multiple sequence alignments were performed using ClustalW with a gap open and gap extension penalties of 10 and 0.1, respectively [70]. The alignment file was then used to construct an unrooted phylogenetic tree based on the neighbor-joining method [71]. After bootstrap analysis for 1000 replicates, the tree was displayed using Interactive tree of life (iTOL; http://itol.embl.de/index.shtml) [72]. Protein sequence motifs were identified using the multiple EM for motif elicitation (MEME); (http://meme.nbcr.net/meme3/meme.html) [73]. The analysis was performed by keeping number of repetitions, any; maximum number of motifs, 20; and optimum width of the motif ≥50. Discovered MEME motifs with ≤1e-30 were searched in the InterPro database with InterProScan [74].

### Gene Ontology (GO) Annotation

The functional annotation of bZIP sequences and the analysis of annotation data were performed using Blast2GO (http://www.blast2go.com) [75]. The amino acid sequences of bZIPs were imported into Blast2GO program to execute three steps viz, (i) BLASTp against the non-redundant protein database of NCBI, (ii) mapping and retrieval of GO terms associated with the BLAST results, and (iii) annotation of GO terms associated with each query to relate the sequences to known protein function. The program provides the output defining three categories of GO classification; namely biological processes, cellular components, and molecular functions.

### Comparative Physical Mapping of bZIP Proteins between Cucumber and other Species

For deriving orthologous relationship among the chromosomes of cucumber and three other species, amino acid sequences of cucumber bZIP were searched against peptide sequences of Arabidopsis, rice and poplar (www.phytozome.net) using BLASTP. Hits with ≤1e-5 and at least 80% identify were considered significant. The comparative orthologous relationships of *bZIP* genes among cucumber, Arabidopsis, rice and poplar chromosomes were finally visualized using MapChart.

### Estimation of Synonymous and Non-synonymous Substitution Rates

The amino-acid sequences, duplicated protein-encoding bZIP genes, as well as orthologous gene-pairs between cucumber and Arabidopsis, rice and poplar were aligned using CLUSTALW based on multiple sequence alignment tool. The CODEML program in PAML interface tool of PAL2NAL (http://www.bork.embl.de/pal2nal) [76] was used to estimate the synonymous (Ks) and non-synonymous (Ka) substitution rates by aligning the amino-acid sequences and their respective original cDNA sequences of *bZIP* genes. Time (million years ago, Mya) of duplication and divergence of each *bZIP* genes were estimated using a synonymous mutation rate of λ substitutions per synonymous site per year, as T = Ks/2λ (λ = 6.5×10 e-9) [77]–[78].

### Homology Modeling of bZIP Proteins

All the CsbZIP proteins were searched against the Protein Data Bank (PDB) [79] by BLASTP (with the default parameters) to identify the best template having similar sequence and known three-dimensional structure. The data was fed in Phyre2 (Protein Homology/AnalogY Recognition Engine; http://www.sbg.bio.ic.ac.uk/phyre2) for predicting the protein structure by homology modeling under ‘intensive’ mode [80].

### Computational Identification of miRNAs Targeting the bZIP Genes

Identification of miRNA-regulated gene targets is crucial for understanding miRNA functions. Previously known plant pre-miRNA sequences obtained from miRBase v20.0 (http://www.mirbase.org) and plant miRNA database (http://bioinformatics.cau.edu.cn/PMRD) were used for identification of miRNAs targeting the *CsbZIP* genes. Therefore, the putative targets of all plant and *C. sativus* miRNAs were identified by aligning all known plant miRNAs with the assembled transcripts of CsbZIPs using the web-based psRNA Target Server (http://plantgrn.noble.org/psRNATarget) with default parameters. Alignment between all known plant miRNA and its potential target(s) was evaluated by the parameters described in Zhang et al. [81]. These computationally identified miRNA targets were further analyzed using BLASTX searches with ≤1e-10 against *C. sativus* EST sequences at NCBI database to identify putative gene homologous for confirmation.

### Expression Profiling of *CsbZIP* Genes using Transcriptome Data

All Illimuna HiSeq reads for RNA-Seq analysis were retrieved from a public repository database (SRA, Sequence Read Archive) following accession numbers; SRR351476 (cucumber ovary tissue, unexpanded), SRR351489 (cucumber expanded ovary tissue, fertilized), SRR351495 (cucumber expanded ovary tissue, unfertilized), SRR351499 (cucumber root tissue), SRR351905 (cucumber stem tissue), SRR351906 (cucumber leaf tissue), SRR351908 (cucumber male flower tissue), SRR351910 (cucumber tendril tissue), SRR351911 (cucumber tendril tissue basal) and SRR351912 (cucumber female flower tissue). All reads were downloaded in raw sequencing data “.sra” format and converted to “fastq” format by the NCBI SRA Toolkit's fastq-dump command. After discarding low-quality reads (Phred quality (Q) score <20) and trimming adapters by using FASTX toolkit, all clean reads were subjected to FastQC analysis for checking read qualities in terms of per-base sequence qualities, per-sequence quality scores, per-base nucleotide content and sequence duplication levels.

Following all preprocessing steps outlined above, the high-quality 75-b pair end reads were aligned to a Bowtie2-indexed *C. sativus* genome (v1.0) using the TopHat alignment software suite (http://tophat.cbcb.umd.edu). In RNA-Seq analysis, read counts have been found to be linearly related to the abundance of the transcripts. Therefore, before estimating read counts, the BAM (aligned read) file generated by TopHat must first be processed by appropriate software tools. SAMtools (v0.1.19) command tool was used for sorting, converting and indexing BAM files. For estimating raw counts, BEDTools (2.16.2) was used to estimate the number of raw reads by calculating mapped reads. The raw count data were normalized using DESeq’s normalization step. Then, a variance-stabilizing transformation (VST) was performed on the normalized gene data set for downstream gene by applications, such as gene, gene expression measurement and hierarchical clustering. A two-way hierarchical clustering heat map was constructed based on these expression values, using euclidean distance metric and average linkage method. Heat maps and hierarchical clustering were computed with the gplots package in R.

### Plant Materials, Growth Conditions, and Treatments

Cucumber seeds (Altay cultivar) were obtained from Monsanto Gıda ve Tarım (Antalya, Turkey). The seed coats were removed, and the seeds were washed with distilled water 3 times. Then, they were transferred to plastic containers and grown in hydroponic culture containing half-strength Hoagland’s Solution [82] for 14 days in a plant growth chamber at 24±2°C with a 16 h light and 8 h dark photoperiod at a light intensity of 400 µmol m^–2 ^s^–1^. For drought stress, 10% polyethylene glycol 6000 (PEG-6000) was added to the half-strength Hoagland’s solution. Stress treatment was initiated on the 14th day of normal growth. Both treated (stress) and non-treated (control) plants were kept in the growth chamber with the same growth conditions. Samples from the treated and control plants were harvested after 0, 3, 12, and 24 h of stress application. Time point zero (0 h) was used as a control. The roots and leaves of mature plants were collected separately and used for tissue-specific expression analysis. The tissues from 3 biological replicates were immediately frozen in liquid nitrogen.

### RNA Extraction and Quantitative Real-time PCR Analysis

Total RNA extraction was performed with TRIzol reagent (Life Technologies Corporation, Grand Island, NY, USA). DNA contamination in samples was removed with DNase I (Fermentas, Thermo Fisher Scientific, Waltham, MA, USA) according to the manufacturer’s instructions. The quality and integrity of the total RNA was checked with agarose gel electrophoresis and the NanoDrop 2000D (NanoDrop Technologies, Wilmington, DE, USA).

For RT-PCR, the specific primers were designed according to the *bZIP* gene sequences by Primer 5 software (http://www.primer-e.com/index.htm) (Table S8). Based on the literature search, highly expressed *bZIP* genes under the drought stress were selected for quantitative real-time-PCR. A cucumber *18S rRNA* gene (GenBank ID: X51542.1), amplified with primers 5′-GTGACGGGTGACGGAGAATT-3′ and 5′- GACACTAATGCGCCCGGTAT-3′, was used as a control. A suitable program was optimized according to primers Tm temperatures. Three biological replicates were carried out and triplicate quantitative assays for each replicate were performed using SYBR Green PCR Master mix kit (Roche Applied Science). The cucumber *18S rRNA* gene was used as an internal control. Relative gene expression was calculated. The ΔCT and ΔΔCT were calculated by the formulas ΔCT = CT target - CT reference and ΔΔCT = ΔCT treated sample - ΔCT untreated sample (0 h treatment). For all chart preparations, selected RNA relative amount was evaluated for gene expression level using the 2- ΔΔCT. At the same time, the standard errors of mean among replicates were calculated. Student’s t-test was used to obtain the statistical significance of the difference between treated samples and untreated samples (0 h treatment under abiotic stress). If P-values <0.01, we considered the *bZIP* genes as differentially expressed genes.

## Results and Discussion

### Genome-wide Identification of the bZIP Gene Family TFs in the Cucumber Genome

To identify *bZIP* TF genes in cucumber, both BLAST and Hidden Markov model (HMM) searches were performed. To better explore their expansion mechanisms, evolutionary history and expression divergence, seven plant genomes including Arabidopsis, grape, maize, papaya, poplar, rice, and sorghum were used for searches. After multiple cycles of searches, a total of 741 putative *bZIP* genes were detected in these plant genomes. These members were then subjected to the Pfam and SMART domain searches to validate the presence of the bZIP-related domains. By removal of different transcripts of the same gene, we identified 64 putative *CsbZIP* genes (Table S1). Both the search outputs showed the presence of bZIP-related domains in all the 64 *CsbZIP* genes. For convenience, the 64 *CsbZIP* genes were named from *CsbZIP-01* to *CsbZIP-64* based on their order on the chromosomes, from chromosomes 1 to 7. Two *bZIP* genes (Cucsa.213060, Cucsa.365420) that could not be conclusively mapped to any chromosome were renamed *CsbZIP-63* and *CsbZIP-64*, respectively.

The *CsbZIP* genes vary substantially in the size and sequences of their encoded proteins, and their physicochemical properties. The location of the bZIP-related domains within the protein also differs. Protein length of CsbZIPs varied from 132 to 721 amino acids. EXPASY analysis suggested that the CsbZIP protein sequences had large variations in isoelectric point (pI) values (ranging from 4.6 to 9.9) and molecular weight (ranging from 15.308 kDa to 78.470 kDa). The details of other parameters of CsbZIP protein sequences were summarized in Table S1.

In Arabidopsis, a total of 75 and 77 *bZIP* gene family members have been identified by Jakoby et al. [10] and Correa et al. [15], respectively. However, Wang et al. [14] detected with the incomplete bZIP domain or lacked this domain based on the Pfam or SMART domain searches. So, they listed 72 members of *bZIP* gene in Arabidopsis [14]. Rice *bZIP* gene family was previously identified on a genome-wide level [13], [15]. Although these two studies reported the presence of 89 *bZIP* genes in rice, Wang et al. [14] made a detailed comparison with the sorghum bZIP family and found 88 bZIP TFs. Due to lack of bZIP domain, they eliminated some of the *bZIP* genes. Also, it has been reported that castor bean [11], maize [12], sorghum [14] and poplar [15] genomes encode 49, 125, 92 and 89 members of the *bZIP* gene family, respectively.

### Chromosomal Distribution and Structure of CsbZIP

**Figure 1:**
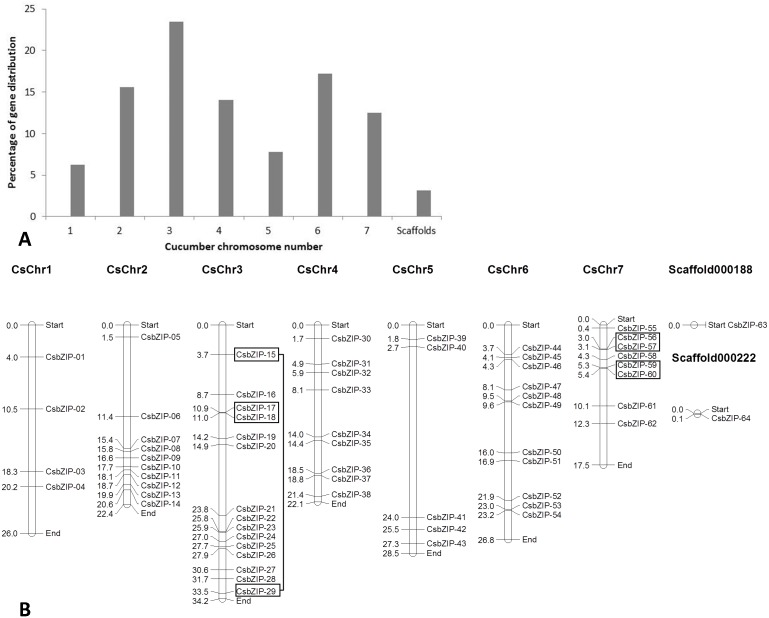
Distribution of 64 *CsbZIP* genes onto seven cucumber chromosomes. (A) Percentage of *bZIP* genes on each cucumber chromosome to show their distribution abundance. (B) Graphical (scaled) representation of physical locations for each *CsbZIP* gene on cucumber chromosomes (numbered 1–7). Tandem-duplicated genes on a particular chromosome are indicated in the box. Chromosomal distances are given in Mbp.

All cucumber *CsbZIP* gene members were physically mapped in all the 7 chromosomes of cucumber. Among all, chromosome 3 contains the highest number of *CsbZIP*s (23.4%), while minimum genes were distributed on chromosome 1 (6.3%) (Figure 1A). The exact position (in bp) of each *CsbZIP* on cucumber chromosome is given in Table S1. Distribution pattern of the *CsbZIP* genes on individual chromosomes also indicated certain physical regions with a relatively higher accumulation of gene clusters. For example, *CsbZIP* genes located on chromosomes 2, 3 and chromosomes 7 appear to be congregate at the lower end and upper end of the arms, respectively (Figure 1B).

Tandem duplication of *CsbZIP* gene members was also determined. Tandem duplicated genes on a particular chromosome were indicated in the box as shown in Figure 1B. Totally, we detected only four pairs of tandem duplicates (Table S2), indicating the limited contribution of tandem duplication to the gene family expansion. A similar result has been observed in the sorghum, rice and Arabidopsis genomes [14]. The distance between these genes ranged from 1 kb to 33.4 kb. We then carried out a genome-wide identification of segmentally duplicated *bZIP* genes in cucumber. Totally, 12 segmental duplicated cucumber *bZIP* genes have been detected, accounting for around 19% (12/64) of total *CsbZIP* genes (Table S3). Segmental duplication has been regarded as a major driver to contribute to the expansion of gene families. Segmental duplication rate of the *bZIP* genes was also examined in other plant species such as Arabidopsis, rice and sorghum, which ranging between 53% and 59% [14]. They showed higher segmental duplication rate, when compared to rate of cucumber *bZIP* genes.

Exon-intron organization of the 64 cucumber *bZIP* genes was also investigated to obtain some insight into their gene structures (Figure S1). We have detected a total of 12 *bZIP* genes with no intron, accounting for 18.75% of total *CsbZIP* genes. Similar cases have also been observed in Arabidopsis, castor bean, rice and sorghum [10]–[11], [13]–[14], suggesting the evolutionary conservation. Most of these intronless genes were clustered into the Cluster I a (Figure S1). Among the intron-containing *bZIP* genes in cucumber, the number of introns in their open reading frames varied from 1 to 12. They were distributed into different classes of the bZIP family. In castor bean, there were 11 introns [11], being 12 in Arabidopsis and rice, [10], [13] and 14 in sorghum [14].

### Phylogenetic Classification of CsbZIPs and Identification of Domain Conservation

**Figure 2:**
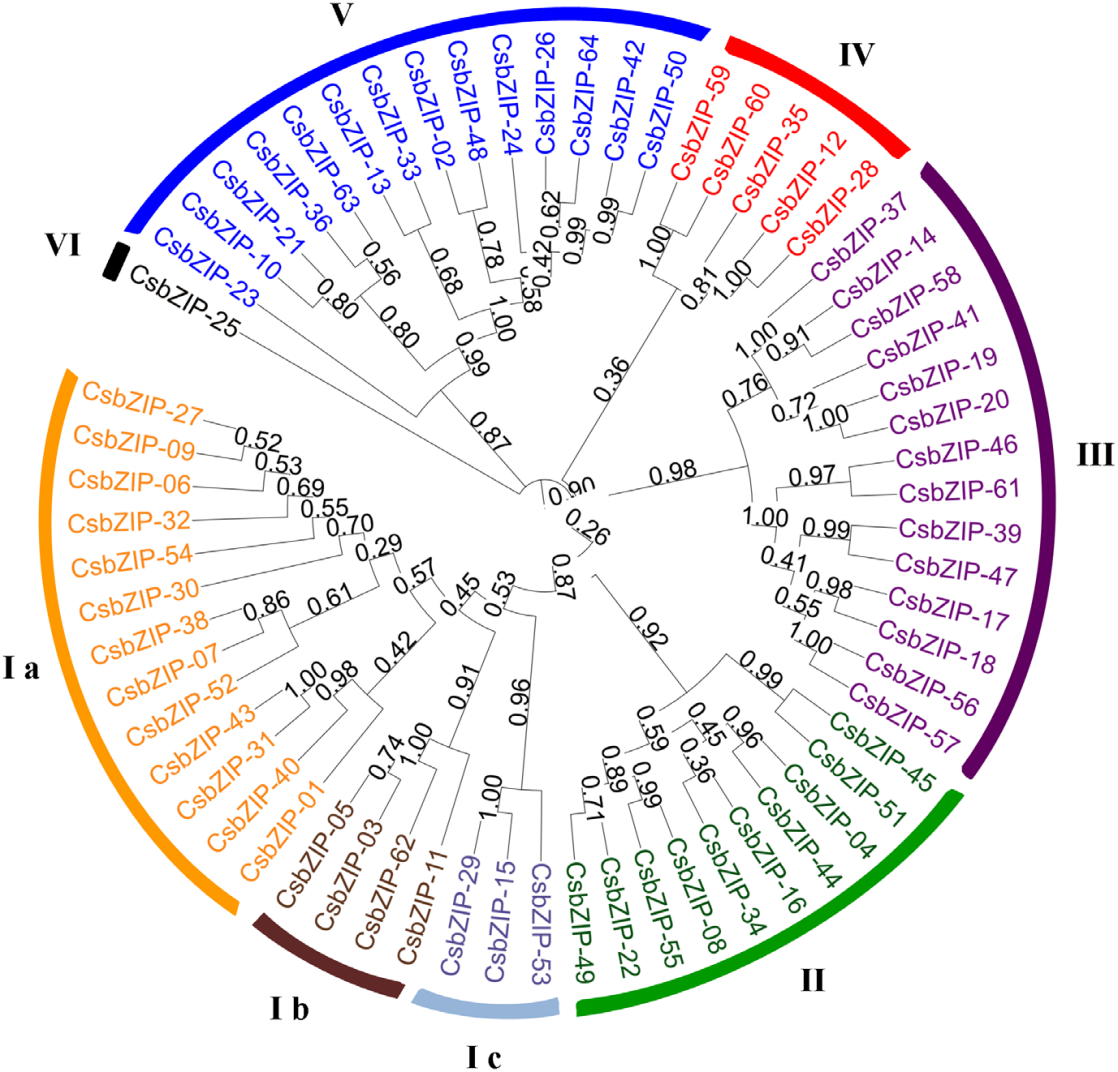
Phylogenetic relationships of cucumber bZIP proteins. The sequences were aligned by CLUSTALW at MEGA5 and the unrooted phylogenetic tree was deduced by neighbor-joining method. The proteins were classified into six distinct clusters. Each family was assigned a different color according to well-known members in other species.

The comprehensive phylogenic analysis was performed to understand the evolutionary significance of domain architecture in CsbZIP proteins. The phylogenetic tree was constructed with 64 CsbZIP proteins by Neighbour-Joining (NJ) method. The phylogenetic analysis categorized all the CsbZIPs into six discrete groups (Cluster I to VI) comprising of 20, 10, 14, 5, 14, and 1 proteins, respectively (Figure 2). Since a good number of the internal branches were observed to have high bootstrap values, it was clearly through bootstrap analysis of 1000 replicates. A good number of the internal branches had high bootstrap values, reflecting derivation of statistically reliable pairs of possible homologous. Phylogeny-based function prediction has been applied for prediction of bZIP proteins in other species like rice, Arabidopsis and soybean. A total of 7 and 10 groups of bZIP TFs have been classified in sorghum and Arabidopsis, respectively. They were named as group A to I and S [10], [14]. Compared with the nomenclature of Arabidopsis and sorghum, both group I and E have been combined into the class 1; class 2 consists of group B, D, F and H; and group S has been divided into classes 6 and 7. In addition, rice OsbZIP transcription factors were subdivided into 10 clades, designated A to J, with well-supported bootstrap values [13]. In a different study, totally 333 sequences were analyzed to indicate phylogenetic relationship of bZIP transcription factors among maize (170 sequences), rice (89 sequences) and Arabidopsis (74 sequences) [12]. The three plant species had some divergences against the rice OsbZIP proteins in the phylogenetic tree. Certain members of groups were separated from their clusters which have also been observed by Nijhawan et al [13]. So, it can be concluded that the interspecies clustering indicates a parallel evolution of bZIP transcription factors in three plants.

Additionally, reliability of the phylogeny was further evidenced by parameters like motif compositions. MEME analysis identified 12 motifs according to their domain compositions of the cucumber bZIPs (Table 1, Figure S2). Except for CsbZIP10, CsbZIP21, CsbZIP36, and CsbZIP63, all CsbZIP proteins contain Basic-leucine zipper domain (Motif 1). Besides bZIP basic domain, two other known functional domains were classified into the CsbZIPs. Fourteen members containing the transcription factor TGA-like domain (Motif 2) were identified as Cluster V in which CsbZIP10, CsbZIP21, CsbZIP36, and CsbZIP63 were found. Eleven members contain CAMP-Response Element Binding Protein (Motif 4). Further, seven unidentified conserved motifs were found. It was observed that a majority of the members, predicted to have similar DNA-binding properties, clustered together. However, certain members of Clusters Ib, III, and V were exceptions because they clustered apart into different clades. All CsbZIP proteins in Cluster V have carried transcription factor TGA like domain. In addition, CsbZIP proteins belonging to Cluster Ib and Cluster III contain an unidentified conserved motif, which was named as Motif 3 (Table 1). Most of the members belonging to one cluster also shared one or more conserved motifs outside the bZIP domain. In addition, 4 tandemly duplicated and 12 segmentally duplicated CsbZIP proteins were located on the same cluster. For example, tandemly duplicated CsbZIP17-CsbZIP18 and CsbZIP56-CsbZIP57 were found in Cluster III; segmentally duplicated CsbZIP proteins (CsbZIP2-CsbZIP48, CsbZIP13-CsbZIP33, CsbZIP26-CsbZIP42, CsbZIP42-CsbZIP50 and CsbZIP50-CsbZIP64) were located on the Cluster V. Such motif-sequence conservation or variation between the proteins specifies the functional equivalence or diversification, respectively, with respect to the various aspects of biological functions [83]. Apart from the bZIP domain region, bZIP proteins usually contain additional conserved motifs, which might indicate potential function sites or participate in activating the function of bZIP proteins. As reported in some earlier studies, diverse conserved motifs outside of the bZIP domain region have been identified in Arabidopsis, castor bean, maize and rice [10]–[13]. Compared to those conserved motifs identified within bZIP transcription factors in other plants, eight motifs (2, 4, 5, 6, 8, 9, 14 and 19) were commonly shared by cucumber, castor bean, Arabidopsis, rice and maize, indicating that these additional motifs outside of bZIP domain might be conserved among plant species. However, some of the other motifs were variable among species and might be species-specific in plants.

**Table 1:** Amino acid composition of the cucumber bZIP motifs.

Motif No.	Sites	E-value	Amino acid sequence composition of motif	Width (aa)	Domain
Motif 1	50	3.4e-771	D[EPQ][KR][RK]Q[RK]R[MIL][IL][SAK]NR[EQ]SA[RA]RSR[LEM]RK[QK][AR][YH][LI]x[EQ]L	29	bZIP_Basic
Motif 2	13	4,90E-201	[SG][KV]AAKAD[VI]FH[LIV][FL][ST]G[MP]WKT[PS]AERC[FL]LW[IL]GGFR[PS]SEL	35	Transcription factorTGA like domain
Motif 3	20	8,60E-265	ER[KQ]VQTLQ[TA]E[AN][TS][TS]LSA[QRE][LV][TA][LFD]L[QS][QR][DQ][TYR][LN]G[LA][TNS][VT][ED]N[SR][EA]LK[LQ]R[LI][QEA][AT][LM][ER][QA][QK][AVK][QH]L[RAK][DE]	50	NA
Motif 4	11	3,30E-148	LE[GS]F[IL]RQAD[NL]LRQQTLQ[QR][MV]HRILTTRQ[AS]ARALLAI[AG][ED]YFSRLRA	44	CAMP-response elementbinding protein
Motif 5	9	1,40E-160	F[DE][MV]EYARW[LV][ED]E[HQ][NHQ]R[LQ][IM][NC][ED]LRAA[VL][NQ][SA]H[LA][SGPT]D[TI]ELRI[IL]V[DE][GSN][CIV][LIM][AT]HYDE[LFIV]FR[LM]K	50	Transcription factorTGA like domain
Motif 6	9	5,40E-133	[LI]K[LIMV]L[VM][PSN]Q[LI][ED][PT]LT[ED]QQ[LI][ML][GE]IC[NK]LQQSSQ[QE][AT]EDALSQG[ML][ED][AKQ]L[QH]Q[SN]L[AIS][ED][TS][LIV][AS][SG]	50	bZIP_Basic
Motif 7	32	1,10E-90	A[EL]N[EA]ALK[EA]E[VI]QRL[KR]x[LA]Lx[QDE]Lx	21	NA
Motif 8	9	1,60E-59	SR[IL]KL[TA]QLEQ[ED]L[QEH]RAR[QS]QG[IL]F	21	NA
Motif 9	6	7,10E-43	GK[DNP][FL]GSMN[ML]DE[LF]LKNIWTAE[EA]NQ[TA][MV]	25	NA
Motif 10	10	6,30E-44	[PT][SRY][FW][RLS][MDV][DN][ISL][SEL][NHKR][ME][PS][ED][NAT][PL][PHRV][RP][RGN][SKV][GHA]HRR[SA][HNS]S[DE][ISTV][SFLP][FAT]	29	NA
Motif 11	7	2,80E-39	[NSY]LQRQ[GA]S[LF][ST]LPR[APT]L[SC][GQ]KTVDEVW[KS][ED]I[HQ]	27	NA
Motif 12	7	2,20E-37	[RS]Q[PQ]TLGE[MV]TLE[DE]FL[IV][KR]AGVV[RA]E	22	NA

### Gene Ontology Annotation

**Figure 3:**
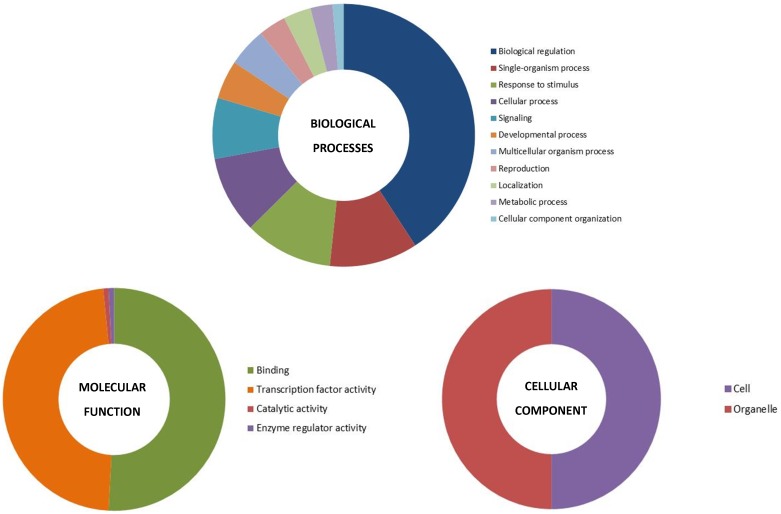
Gene Ontology (GO) distributions for the bZIP proteins. The Blast2Go program defines the gene ontology under three categories (A) biological processes, (B) molecular functions and (C) cellular component.

The GO slim analysis conducted through Blast2Go showed the putative participation of 64 CsbZIP proteins in diverse biological processes (Figure 3, Table S4). Of the 11 categories of biological processes defined by Blast2Go, predominant of CsbZIPs were predicted to function in response to biological regulation (∼41%) [60], followed by single-organism process and response to stimulus (∼11%) [16]. Molecular-function prediction showed that about 64 (∼51%) CsbZIP were evidenced to participate in small molecule binding, which concords with the molecular role of bZIP proteins in assisting protein-protein interactions. Regarding molecular function, about 60 (∼48%) CsbZIP showed transcription factor activity, which correlates with abiotic stress tolerance behavior of cucumber. Cellular localization prediction showed that 17 (∼50%) CsbZIP proteins are localized in the cell part, of which 17 (∼50%) are organelle-localized (Figure 3; Table S4).

### Orthologous Relationships of *bZIP* Genes between Cucumber and other Species

For comparative mapping to derive orthologous relationships of CsbZIPs, the physically mapped *CsbZIP* genes were compared with those in chromosomes of other related genomes, namely Arabidopsis, rice and poplar. (Table 2, Figure S3). Of the identified 64 CsbZIP protein-encoding genes in cucumber, the specific orthologous relationships could be derived on an average for ∼73.3% proteins. Maximum orthology of *CsbZIP* genes annotated on the cucumber chromosomes was obtained with poplar (91%), followed by Arabidopsis (73%) and rice (56%). The extensive gene-level synteny shared among cucumber, poplar and Arabidopsis supports their close evolutionary relationships. Interestingly, most of *CsbZIP* genes revealed syntenic biases towards particular chromosomes of Arabidopsis and poplar. For instance, maximum orthology was obtained between *CsbZIP* genes on cucumber chromosome 2 and Arabidopsis chromosome 1 (90%). In addition, the *CsbZIP* genes on cucumber chromosome 7 showed 88% orthology and colinearity with Arabidopsis chromosome 2 and poplar chromosome 4 (75%) (Table 2). A similar result was observed between Arabidopsis, rice and sorghum [14]. Total 72 sorghum bZIP genes could find their corresponding 66 rice orthologs. Also, six Arabidopsis *bZIP* genes were detected with seven orthologs in the rice genome [14]. The results indicated that chromosomal rearrangements like duplications and inversions were predominant in shaping the distribution and organization of *CsbZIP* genes in cucumber, Arabidopsis, rice and poplar genomes. The information from comparative mapping provides a useful preface for understanding the evolutionary process of *bZIP* genes among cucumber and other plant species. This can be also useful for isolation of orthologous *bZIP* genes from cucumber, using the map-based genomic information of other related plant-species for genetic enhancement.

**Table 2:** A summary of comparative mapping of cucumber *bZIP* genes on Arabidopsis, rice and poplar.

*Cucumis sativus*	*Arabidopsis thaliana*	*Oryza sativa*	*Populus trichocarpa*
**Chr 1 (4)**	Chr 5 (50%)	-	-
**Chr 2 (10)**	Chr 1 (90%)	Chr 2 (40%)	Chr 5 (30%)
**Chr 3 (15)**	Chr 1 (73%) Chr 7 (46%)	Chr 12 (27%) Chr 9 (20%)	Chr 6 (40%)
**Chr 4 (9)**	Chr 3 (55%)	-	Chr 2 (33%)
**Chr 5 (5)**	Chr 5 (60%)	Chr 3 (40%) Chr 7 (40%)	Chr 13 (40%)
**Chr 6 (11)**	Chr 3 (46%)	Chr 1 (45%)	Chr 5 (27%)
**Chr 7 (8)**	Chr 2 (88%) Chr 4 (50%)	Chr 2 (25%) Chr 6 (25%)	Chr 4 (75%) Chr 9 (50%)
**Scaffolds (2)**	-	-	-
**Organism (64)**	73%	56%	91%

### Duplication and Divergence Rate of the *CsbZIP* Genes

**Figure 4:**
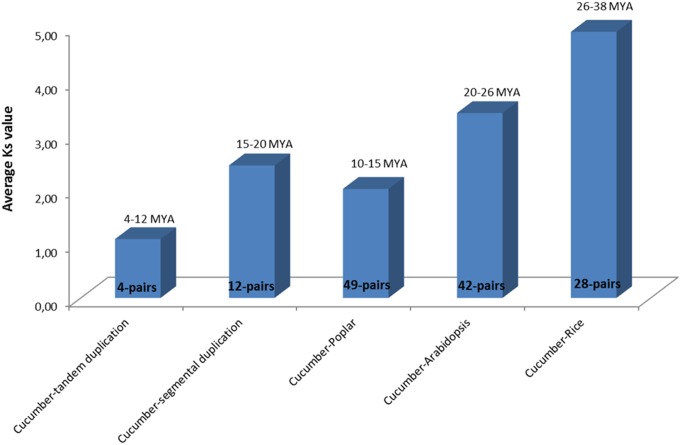
Time of duplication and divergence (MYA) based on synonymous substitution rate (Ks), which estimated using duplicated *bZIP* gene pairs of cucumber and orthologous *bZIP* gene pairs between cucumber and poplar (49) or Arabidopsis (42) or rice (28).

Multiple copies of genes in a gene family could possibly evolve due to the flexibility provided by events of whole-genome tandem and segmental duplications. Gene duplication, either segmental or tandem, has been documented in several plant TF gene-families such as NAC, MYB, F-box, as well as in bZIP [12]–[14], [83]–[85]. We thus explored association of Darwinian positive selection in divergence and duplication of *bZIP* genes to understand the expansion of this gene family. To interpret this, the ratios of non-synonymous (Ka) versus synonymous (Ks) substitution rates (Ka/Ks) were estimated for four tandem and 12 segmentally duplicated gene-pairs, as well as between orthologous gene-pairs of *CsbZIP* with those of poplar (49-pairs), Arabidopsis (42) and rice (28). The ratios of Ka/Ks for tandem duplication varied from 0.09 to 0.20 with an average of 0.13 (Table S2), whereas Ka/Ks for segmentally duplicated gene-pairs ranged from 0.04 to 0.23 with an average of 0.11 (Table S3). It suggested that the duplicated *CsbZIP* genes are under strong purifying selection pressure, since their Ka/Ks ratios were estimated as <1. Additionally, the duplication event of these tandemly and segmentally duplicated genes may be estimated to have occurred around 4–12 and 15–20 Mya, respectively (Figure 4). Among the orthologous gene-pairs of CsbZIP with those of other plant species, the average Ka/Ks values were maximum between poplar and cucumber (0.14) and minimum for Arabidospsis and rice-cucumber gene-pairs (0.10; Table S5). Although synonymous substitution rates between rice-cucumber and Arabidopsis-cucumber bZIP genes were the same, the earlier divergence was observed from rice-cucumber around 26–38 Mya, as compared to Arabidopsis-cucumber *bZIP* genes (20–26 Mya). Conversely, the *bZIP* gene-pairs between poplar and cucumber seem to have largely encountered intense purifying selection, as compared to rice-cucumber and Arabidopsis-cucumber *bZIP* genes. It agreed well with their recent time of divergence around 10–15 Mya (Figure 4). The estimation of tandem and segmental-duplication time (average of 8.3 Mya and 18.8 Mya, respectively) of cucumber *bZIP* genes in between the divergence time of cucumber-rice (37.8 Mya) and -Arabidopsis (26.2 Mya) and -poplar (15.5 Mya) orthologous *bZIP* gene-pairs are comparable to evolutionary studies, involving the protein-coding genes annotated from the recently released draft genome sequences of cucumber [1]. Interestingly, the *CsbZIP* gene-pairs showing tandem and segmental duplication events are under similar evolutionary pressure (Ka/Ks = 0.13, Ka/Ks = 0.11, respectively), of which the tandemly-duplicated genes revealed much recent duplication events (average 8.3 Mya), in contrast to that estimated for segmentally duplicated gene-pairs (average 18.8 Mya). It overall suggests that the segmental and tandem duplication events have played a predominant role in evolution, for shaping such gene family in foxtail millet. A total of three pairs of tandem duplicates were detected in the sorghum and a similar result have been also observed in the rice and Arabidopsis genomes [14]. Genome-wide analysis of segmentally-duplicated *bZIP* genes showed that in rice, sorghum and Arabidopsis, a total of 52, 49 and 39 *bZIP* genes have been also detected, respectively [10], [13]–[14]. It can be concluded that these segmentally-duplicated genes have contributed to the expansion of multiple classes of *bZIP* genes in different plant species.

### Identification of miRNAs Targeting CsbZIP Transcripts

Two important parameters have been taken into consideration for identification of targets. To score the complementarity between miRNA and their target transcript, we have applied the scoring schema according to miRU [81]. The maximum expectation is the first one, which is the threshold of the score. A miRNA/target site pair has been discarded if its score is greater than the threshold. The default cut-off threshold was adjusted to 3.0. The second one is an UPE defining as maximum energy to unpair the target site. The accessibility of mRNA target site to miRNA has been identified as one of important factors that are involved in target recognition. The psRNATarget server employs RNA to calculate target accessibility, which is represented by the energy required to open (unpair) secondary structure around target. The less energy means the more possibility that small RNA is able to contact (and cleave) target mRNA.

A total of 21 *CsbZIP* genes (CsbZIP4-8-13-15-20-22-23-24-37-39-40-45-46-47-48-49-52-53-55-56-57) targeted by 38 plant miRNAs were identified in cucumber by psRNATarget: A Plant Small RNA Target Analysis Server. However, some plant miRNAs could not indicate any gene target. Among the target genes, *CsbZIP20* and *CsbZIP22* are the most abundant transcripts, which were targeted by all 38 plant miRNAs (Table S6). According to the results of BLASTX analysis of the identified miRNA targets, many of the targets were homologous to conserved target genes existing in other plants species; these targets included bZIP transcription factors (also called as abscisic acid-insensitive 5-like protein, transcription factor TGA7-like, transcription factor RF2b-like) transcription factor HBP-1a-like, G-box-binding factor 4-like, transcription factor VIP1-like, TGACG-sequence-specific DNA-binding protein and some uncharacterized proteins. Most of these targets were found to be responsible for plant growth and response to environmental changes. For example, the target transcript of miR166 was ABA-insensitive gene (*ABI*5), which functions in plant development [86] and in response to stress stimulus, such as NaCl, drought, ABA and cold stress in Arabidopsis [87].

### Homology Modeling of CsbZIP Proteins

**Figure 5:**
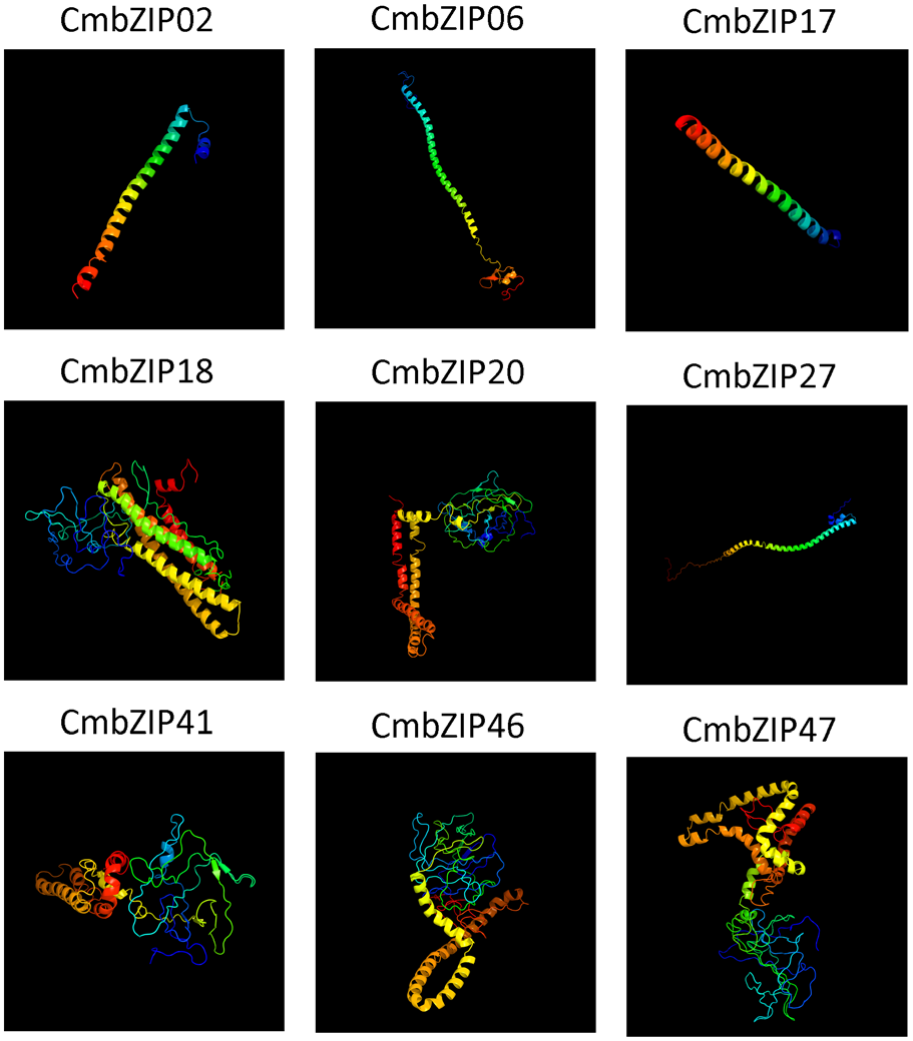
Predicated structures of bZIP proteins. The structure of 9 bZIP proteins with >90% confidence level are shown.

BLASTP search was performed against the PDB in order to build the homology model. Nine CsbZIP proteins (CsbZIP2-6-17-18-20-27-41-46-47) having higher homology were selected. Detection rate was utilized for prediction of homology modeling in Phyre2, which uses the alignment of hidden Markov models via HMM-HMM search [88] to significantly improve the accuracy of alignment. The intensive mode of Phyre 2 uses the multi-template modeling for higher accuracy. Furthermore, it integrates a new *ab initio* folding simulation termed as Poing [89] to model regions of proteins with no noticeable homology to known structures. The protein structure of all the nine CsbZIP are modelled at >90% confidence and the percentage residue varied from 80 to 100 (Figure 5). The secondary structure predominantly comprised of α helices, with rare occurrence of β sheets. Hence, all the predicted protein structures are considered highly reliable, and this offers a preliminary basis for understanding the molecular function of CsbZIP proteins.

### Genome-wide Tissue-specific Expression Profile of bZIP Transcription Factors

**Figure 6:**
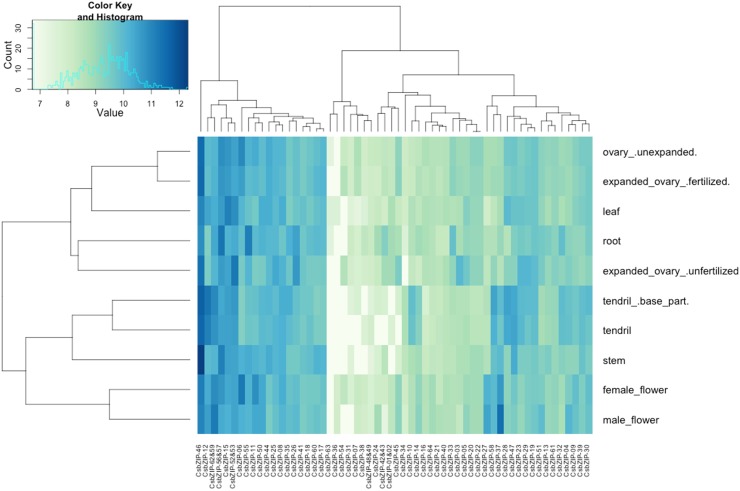
Heatmap of the differentially expressed *CsbZIP* genes in different tissues. The image summarizes the tissue-specific expression pattern of 64 bZIP transcription factors. Note that expression values mapped to a color gradient from low (plain green) to high expression (dark blue).

**Figure 7:**
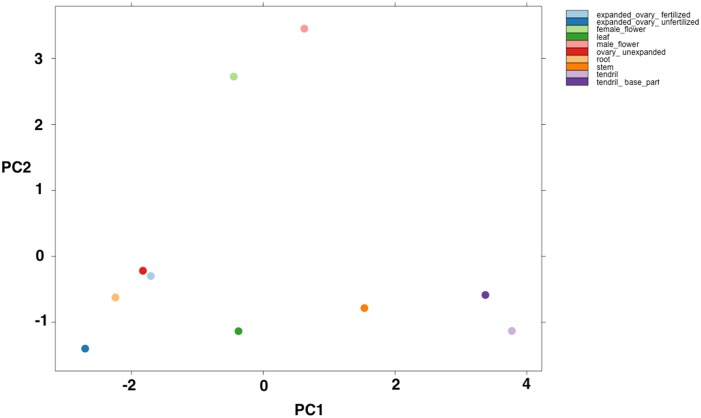
PCA score plots of different tissues. The graph shows a clear separation between flower and other tissues.

One of the main purposes of a gene expression profiling on a genomic scale is to determine those genes that are differentially expressed within the organism being studied. Thus, to gain insight into the tissue-specific gene expression patterns of bZIP transcription factors in cucumber, a RNA-Seq approach was applied to data sets obtained from SRA. After normalization using DEseq and variance-stabilizing transformation for gene expression, *bZIP* genes were ranked from highest to lowest according to their differential expression across main tissues. Hierarchically clustered heat map in Figure 6 showed that *CsbZIP-46* gene was highly expressed in ovary (unexpanded, unfertilized and fertilized), stem, tendril, tendril base tissues, whereas *CsbZIP-06*, *CsbZIP-15*, *CsbZIP-37* and *CsbZIP-56/57* genes were found to be highly expressed in female flower, leaf, male flower and root tissues, respectively. Moreover, while *CsbZIP-36* and *CsbZIP-63* genes had their lowest expression level in reproductive tissues (all ovary tissues and female flower), the lowest expression level of *CsbZIP-54* was observed particularly in vegetative tissues (root, stem, leaf and tendril) and male flower. But when considering all of *CsbZIP* genes with expression level, we calculated and compared the coefficient of variation (CV) of *bZIP* genes for determining the most variable expression phenotypes among the tissues (Table S7). Although *CsbZIP-54* exhibited very low expression in many tissues (Figure 6), its expression was most variable among tissues according to the coefficient of variation value, contrary to *CsbZIP-61*, *CsbZIP-18* and *CsbZIP-41* whose expressions showed minimal variation among tissues. According to the dendrogram (hierarchical clustering of 64 *CsbZIP* genes) above the heat maps, the expression level of *CsbZIP-27*, *CsbZIP-58* and *CsbZIP-37* genes in female flower tissue showed remarkable similarity to those of *CsbZIP* genes in male flower. These transcription factors might exhibit a unique expression pattern in these reproductive tissues. Moreover, PCA score plots (Figure 7) and dendrogram (left side of the heat map) also showed a clear separation between flower and other tissues. Despite the partial similarity among genes, *CsbZIP* TFs have also a different expression pattern in ovary tissue of cucumber. Namely, the *CsbZIP* TFs from unexpanded ovary shared a higher degree of similarity with ovary-fertilized tissues than with ovary-unfertilized tissue in terms of gene expression level. Therefore, unexpanded ovary and fertilized ovary were clustered together in both PCA-space and dendrogram.

### Drought-responsive *CsbZIP* Genes

**Figure 8:**
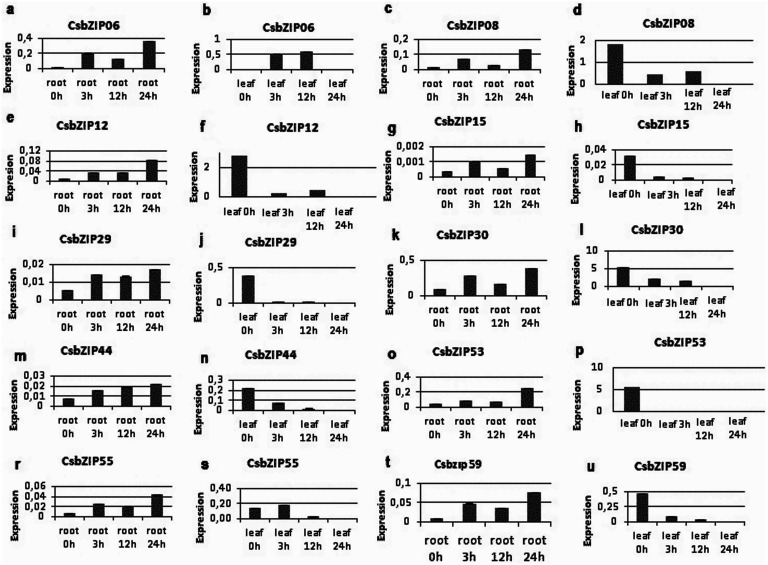
Expression profiles of selected cucumber *bZIP* genes under drought stress. Relative expression levels of the genes upon 0, 3, 12, and 24(a, c, e, g, i, k, m, o, r and t) and leaf (b, d, f, h, j, l, n, p, s and u) tissues are shown.

To identify the drought-responsive cucumber bZIP transcription family members, the qRT-PCR analyses were performed. Expression levels of 10 distinct bZIP family members (*CsbZIP06, CsbZIP08, CsbZIP12, CsbZIP15, CsbZIP29, CsbZIP30, CsbZIP44, vbZIP53, CsbZIP55, CsbZIP59*) were measured in the drought-stressed cucumber leaves and roots. It was observed that all ten selected genes were up-regulated in cucumber roots upon 24 h PEG treatment. Among them, expression of the *CsbZIP12* and *CsbZIP44* showed gradual induction in root tissues with progressing drought stress condition. On the contrary, all selected *CsbZIP* genes were measured as down-regulated in drought-stressed leaf tissue. The expression levels of *CsbZIP29, CsbZIP30* and *CsbZIP44* genes were observed as gradually decreased with increasing drought exposure. Other measured *CsbZIP* family genes displayed various expression levels between 3 and 12 h of drought stress (Figure 8). According to the qRT-PCR results, the cucumber bZIP transcription factors were notably affected and showed changing expression levels in response to water deficiency in tissue-specific manner. Especially, relatively rapid increase in the accumulation of the bZIP transcripts in roots and suppression in leaf tissues were observed upon water deficiency. Such stresses as drought cause to huge crop looses all around the world. Therefore, understanding the plant responses at molecular level is crucial to improve the stress tolerance and productivity. The bZIP TFs are found in all eukaryotes, being considered as one of the largest families of TFs in plants, having diverse responsibilities such as abiotic stress response, seed maturation, flower development and pathogen defense [90]. However, it is not fully understood how they cope with abiotic stresses, particularly tolerating drought. Therefore, several functional studies have been done on the role of bZIP TFs against water-deficit stresses [91]–[93]. In this study, we measured the expression of ten *CsbZIP* TF genes to analyze their possible drought-responsive roles. The accumulation of the measured CsbZIP transcripts in root tissue was observed, whereas the suppression was detected for all of them in leaf upon water deficiency. It supports the general idea that the signals for water deficiency are generally realized first in roots [94]. Differential expression profiles of the *bZIP* genes under drought stress suggest that some other genes functioning in water deficiency might also be regulated by this family. In other biotic and abiotic stress studies, similar results were reported [93], [95].

## Supporting Information

Figure S1 **Exon-intron organization of 6 classes of cucumber** ***bZIP*** **genes.** The bZIP family was classified according to Figure 2. The values in parentheses indicate the number of correponding classes of *bZIP* genes. Exons and introns are represented by green boxes and black lines, respectively.
(TIF)

Figure S2 **Variation in motif clades for the bZIP proteins.** The MEME motifs are shown as differently-colored boxes at the N-terminal and C-terminal region for the transcription regulatory region.
(TIF)

Figure S3 **Comparative physical mapping revealed high degree of orthologous relationships of** ***bZIP*** **genes located on seven chromosomes of cucumber with (A) Arabidopsis, (B) rice and (C) poplar.**
(TIF)

Table S1 **A catalog of 64** ***Cucumis sativus*** **bZIP proteins.**
(XLSX)

Table S2 **The Ka/Ks ratios and estimated divergence-time for tandemly-duplicated bZIP proteins.**
(DOC)

Table S3 **The Ka/Ks ratios and estimated divergence time for segmentally-duplicated bZIP proteins.**
(DOC)

Table S4 **Blast2Go annotation details of bZIP protein sequences.**
(XLS)

Table S5 **The Ka/Ks ratios and estimated divergence time for orthologous bZIP proteins between cucumber, Arabidopsis, rice and poplar.**
(DOCX)

Table S6 **miRNA targets identified by psRNATarget.**
(XLSX)

Table S7 **Expression patterns of the** ***CsbZIP*** **genes analyzed from eight RNA-Seq libraries.**
(XLSX)

Table S8 **List of primers used in quantitative real-time-PCR expression analysis of bZIP genes.**
(DOC)
