# Supplementary figures and images for: Genome-Wide Analysis of the bZIP Transcription Factors in Cucumber
Source: PLoS One. 2014 Apr 23;9(4):e96014. doi: 10.1371/journal.pone.0096014 (PMC3997510; doi:10.1371/journal.pone.0096014)

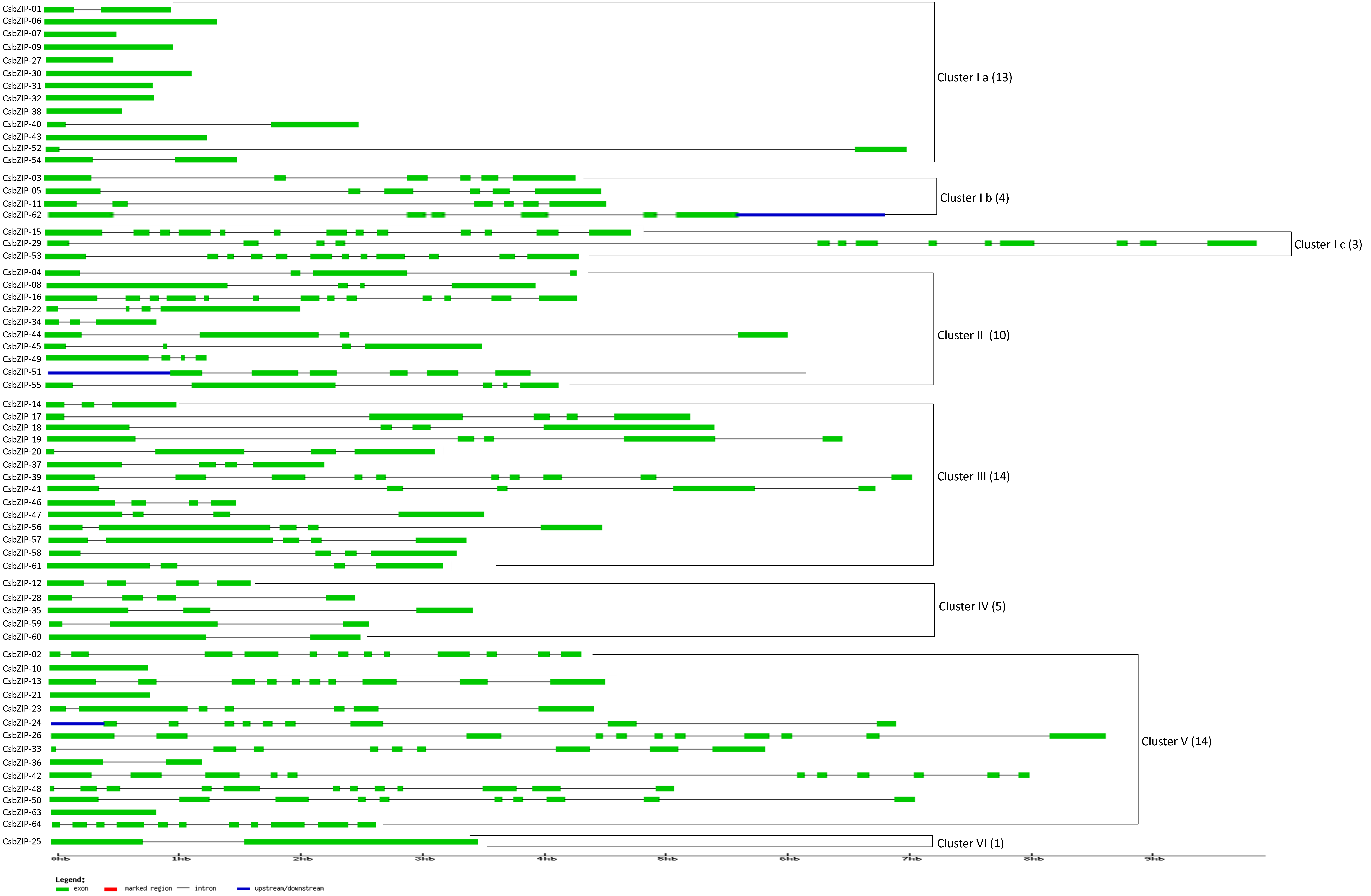

Supplement: Figure S1 — Exon-intron organization of 6 classes of cucumber bZIP genes. The bZIP family was classified according to Figure 2. The values in parentheses indicate the number of correponding classes of bZIP genes. Exons and introns are represented by green boxes and black lines, respectively. (TIF) [file pone.0096014.s001.tif]

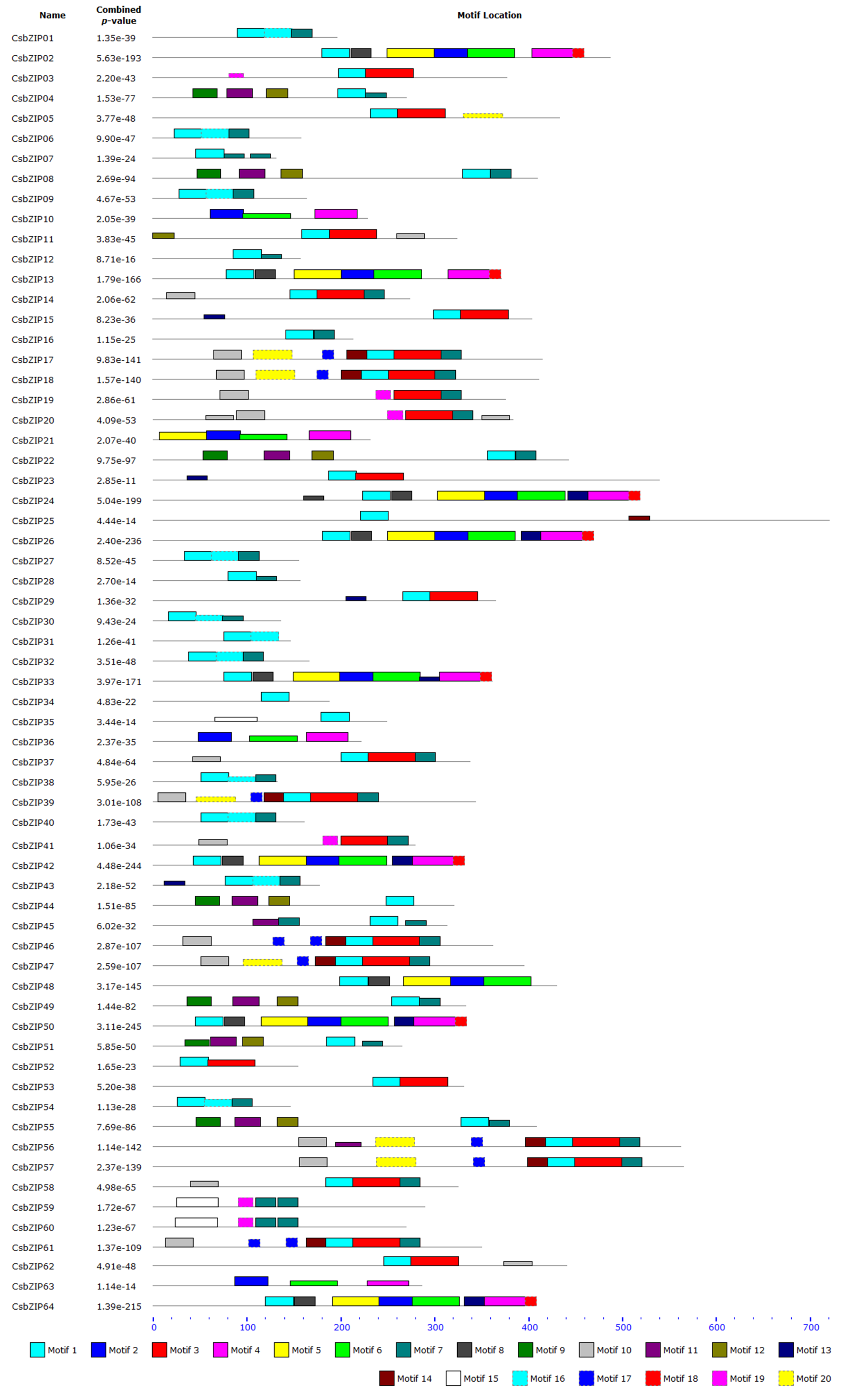

Supplement: Figure S2 — Variation in motif clades for the bZIP proteins. The MEME motifs are shown as differently-colored boxes at the N-terminal and C-terminal region for the transcription regulatory region. (TIF) [file pone.0096014.s002.tif]

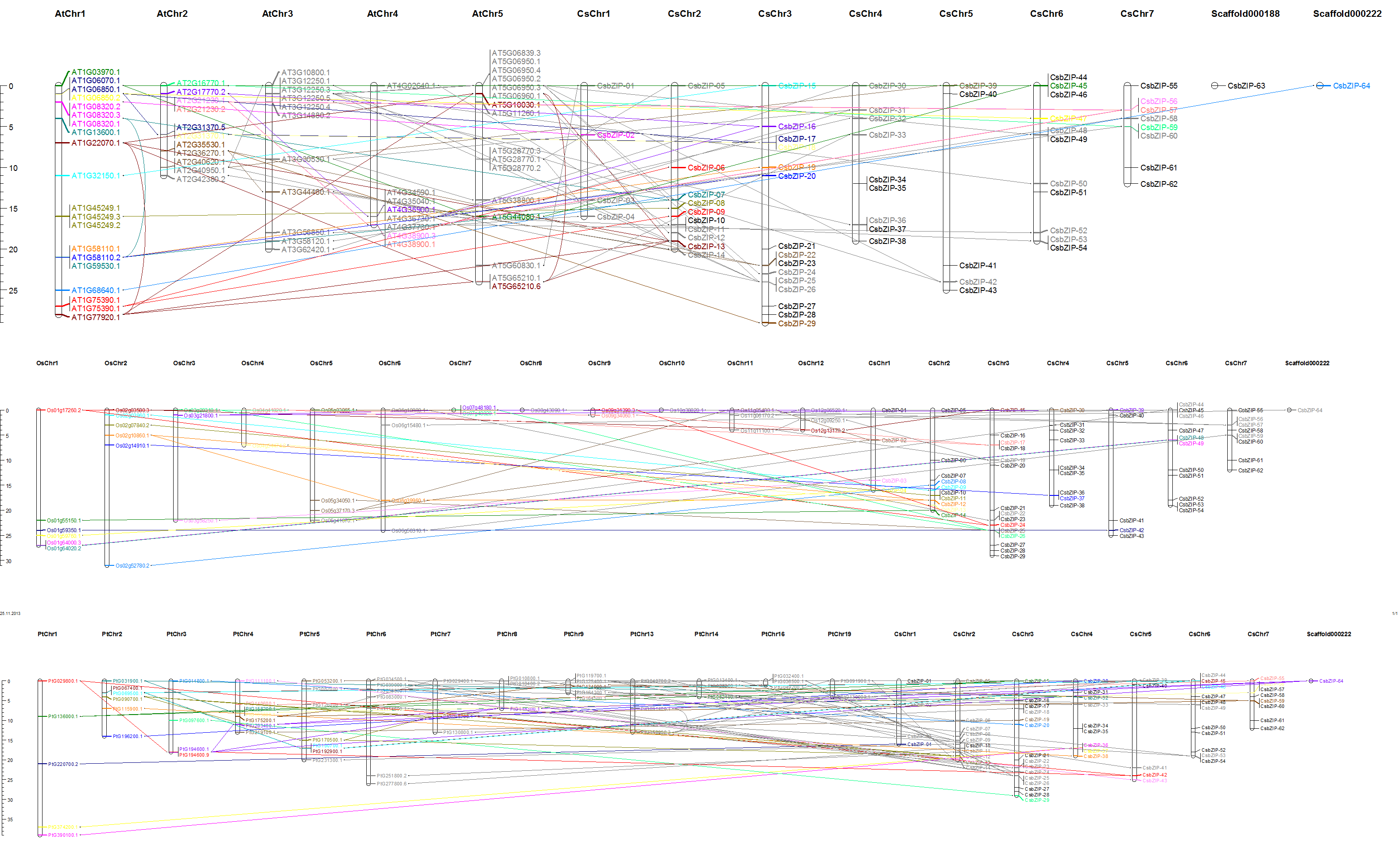

Supplement: Figure S3 — Comparative physical mapping revealed high degree of orthologous relationships of bZIP genes located on seven chromosomes of cucumber with (A) Arabidopsis, (B) rice and (C) poplar. (TIF) [file pone.0096014.s003.tif]
