# Supplementary material for: Genome-Wide Analysis of the bZIP Transcription Factors in Cucumber
Source: PLoS One. 2014 Apr 23;9(4):e96014. doi: 10.1371/journal.pone.0096014 (PMC3997510; doi:10.1371/journal.pone.0096014)
Supplement: Table S2 — The Ka/Ks ratios and estimated divergence-time for tandemly-duplicated bZIP proteins. (DOC) [file pone.0096014.s005.doc]

**Table S2.** The Ka/Ks ratios and estimated divergence time for tandemly-duplicated bZIP proteins.

| **Chromosome** | **Gene** | **Duplicate** | **E value** | **Homology (%)** | **Distance** | **Ks** | **Ka** | **Ka/Ks** | **Mya** |
| --- | --- | --- | --- | --- | --- | --- | --- | --- | --- |
| 3 | CsbZIP-15 | CsbZIP-29 | 1.00E-117 | 77% | 33.456 kb | 1.56 | 0.17 | 0.11 | 12.0 |
| 3 | CsbZIP-17 | CsbZIP-18 | 1.00E-134 | 83% | 0.091 kb | 0.57 | 0.11 | 0.20 | 4.4 |
| 7 | CsbZIP-56 | CsbZIP-57 | 0 | 81% | 0.012 kb | 0.43 | 0.06 | 0.13 | 3.3 |
| 7 | CsbZIP-59 | CsbZIP-60 | 1.00E-135 | 89% | 1.040 kb | 1.79 | 0.16 | 0.09 | 13.8 |
| **Mean** | | | | | | **1.09** | **0.12** | **0.13** | **8.35** |
