# Supplementary material for: Genome-Wide Analysis of the bZIP Transcription Factors in Cucumber
Source: PLoS One. 2014 Apr 23;9(4):e96014. doi: 10.1371/journal.pone.0096014 (PMC3997510; doi:10.1371/journal.pone.0096014)
Supplement: Table S3 — The Ka/Ks ratios and estimated divergence time for segmentally-duplicated bZIP proteins. (DOC) [file pone.0096014.s006.doc]

**Table S3.** The Ka/Ks ratios and estimated divergence time for segmentally-duplicated bZIP proteins.

| **Gene 1** | **Locus** | **Chr.** | **Gene 2** | **Locus** | **Chr.** | **E value** | **Homology (%)** | **Ks** | **Ka** | **Ka/Ks** | **Mya** |
| --- | --- | --- | --- | --- | --- | --- | --- | --- | --- | --- | --- |
| CsbZIP-02 | 10462313-10464935 | 1 | CsbZIP-48 | 9546588-9551391 | 6 | 1.00E-132 | 70% | 1.98 | 0.31 | 0.16 | 15.3 |
| CsbZIP-03 | 18336825-18340804 | 1 | CsbZIP-05 | 1464513-1468666 | 2 | 1.00E-84 | 60% | 2.00 | 0.35 | 0.17 | 15.4 |
| CsbZIP-08 | 15777487-15780470 | 2 | CsbZIP-55 | 435777-438427 | 7 | 1.00E-79 | 56% | 3.45 | 0.35 | 0.10 | 26.6 |
| CsbZIP-12 | 18676801-18678024 | 2 | CsbZIP-28 | 31671425-31687772 | 3 | 3.00E-31 | 69% | 2.79 | 0.19 | 0.07 | 21.4 |
| CsbZIP-13 | 19888987-19892935 | 2 | CsbZIP-33 | 8070029-8074243 | 4 | 1.00E-97 | 66% | 3.62 | 0.32 | 0.09 | 27.9 |
| CsbZIP-14 | 20583871-20585505 | 2 | CsbZIP-58 | 4293029-4296176 | 7 | 2.00E-69 | 64% | 2.75 | 0.32 | 0.12 | 21.2 |
| CsbZIP-26 | 27852009-27859916 | 3 | CsbZIP-42 | 25547501-25554004 | 5 | 1.00E-124 | 84% | 2.63 | 0.10 | 0.04 | 20.2 |
| CsbZIP-31 | 4868223-4868708 | 4 | CsbZIP-43 | 27293844-27294380 | 5 | 5.00E-56 | 82% | 1.24 | 0.18 | 0.14 | 9.5 |
| CsbZIP-38 | 21372791-21373249 | 4 | CsbZIP-07 | 15407143-15407604 | 2 | 8.00E-26 | 65% | 1.70 | 0.39 | 0.23 | 13.1 |
| CsbZIP-42 | 25547501-25554004 | 5 | CsbZIP-50 | 15958340-15962228 | 6 | 1.00E-129 | 77% | 1.04 | 0.07 | 0.06 | 8.0 |
| CsbZIP-46 | 4258111-4259887 | 6 | CsbZIP-61 | 10055463-10058145 | 7 | 5.00E-82 | 68% | 2.66 | 0.28 | 0.10 | 20.5 |
| CsbZIP-50 | 15958340-15962228 | 6 | CsbZIP-64 | 62424-65065 | Scaffold000222 | 1.00E-100 | 71% | 3.48 | 0.20 | 0.06 | 26.8 |
| **Mean** | | | | | | | | **2.45** | **0.25** | **0.11** | **18.8** |
