# Supplementary material for: Genome-Wide Analysis of the bZIP Transcription Factors in Cucumber
Source: PLoS One. 2014 Apr 23;9(4):e96014. doi: 10.1371/journal.pone.0096014 (PMC3997510; doi:10.1371/journal.pone.0096014)
Supplement: Table S5 — The Ka/Ks ratios and estimated divergence time for orthologous bZIP proteins between cucumber, Arabidopsis, rice and poplar. (DOCX) [file pone.0096014.s008.docx]

**Table S5.** The Ka/Ks ratios and estimated divergence time for orthologous bZIP proteins between cucumber, Arabidopsis, rice and poplar.

| **Cucumber -Arabidopsis** | | | | | | | |
| --- | --- | --- | --- | --- | --- | --- | --- |
| **ID** | **Chromosome** | **Gene IDs** | **Chromosome** | **Ks** | **Ka** | **Ka/Ks** | **Mya** |
| CsbZIP-02 | 1 | AT1G08320.2 | 1 | 3.10 | 0.25 | 0.08 | 23.9 |
| CsbZIP-03 | 1 | AT5G28770.2 | 5 | 3.69 | 0.42 | 0.11 | 28.4 |
| CsbZIP-04 | 1 | AT3G56850.1 | 3 | 3.63 | 0.40 | 0.11 | 27.9 |
| CsbZIP-05 | 2 | AT5G28770.3 | 5 | 4.20 | 0.32 | 0.08 | 32.3 |
| CsbZIP-07 | 2 | AT1G59530.1 | 1 | 2.04 | 0.52 | 0.26 | 15.7 |
| CsbZIP-08 | 2 | AT1G45249.1 | 1 | 3.18 | 0.25 | 0.08 | 24.4 |
| CsbZIP-11 | 2 | AT4G02640.1 | 4 | 4.65 | 0.92 | 0.20 | 35.8 |
| CsbZIP-12 | 2 | AT5G11260.1 | 5 | 2.57 | 0.12 | 0.05 | 19.8 |
| CsbZIP-13 | 2 | AT1G77920.1 | 1 | 2.96 | 0.29 | 0.10 | 22.8 |
| CsbZIP-14 | 2 | AT2G42380.2 | 2 | 4.12 | 0.32 | 0.08 | 31.7 |
| CsbZIP-15 | 3 | AT2G35530.1 | 2 | 1.86 | 0.23 | 0.13 | 14.3 |
| CsbZIP-16 | 3 | AT2G17770.2 | 2 | 5.85 | 0.72 | 0.12 | 45.0 |
| CsbZIP-17 | 3 | AT1G06070.1 | 1 | 2.20 | 0.22 | 0.10 | 16.9 |
| CsbZIP-18 | 3 | AT1G06070.1 | 1 | 2.10 | 0.22 | 0.11 | 16.1 |
| CsbZIP-19 | 3 | AT1G58110.1 | 1 | 2.32 | 0.32 | 0.14 | 17.8 |
| CsbZIP-20 | 3 | AT1G58110.1 | 1 | 2.92 | 0.34 | 0.12 | 22.4 |
| CsbZIP-24 | 3 | AT5G06839.3 | 5 | 3.74 | 0.19 | 0.05 | 28.7 |
| CsbZIP-25 | 3 | AT2G40950.1 | 2 | 3.92 | 0.35 | 0.09 | 30.1 |
| CsbZIP-26 | 3 | AT3G12250.5 | 3 | 2.43 | 0.20 | 0.08 | 18.7 |
| CsbZIP-28 | 3 | AT5G11260.1 | 5 | 4.77 | 0.21 | 0.05 | 36.7 |
| CsbZIP-29 | 3 | AT2G35530.1 | 2 | 1.93 | 0.29 | 0.15 | 14.8 |
| CsbZIP-31 | 4 | AT5G38800.1 | 5 | 2.68 | 0.44 | 0.16 | 20.6 |
| CsbZIP-32 | 4 | AT4G34590.1 | 4 | 6.18 | 0.42 | 0.07 | 47.5 |
| CsbZIP-33 | 4 | AT5G10030.1 | 5 | 2.26 | 0.21 | 0.09 | 17.4 |
| CsbZIP-34 | 4 | AT3G56850.1 | 3 | 3.59 | 0.71 | 0.20 | 27.6 |
| CsbZIP-37 | 4 | AT3G58120.1 | 3 | 3.18 | 0.38 | 0.12 | 24.5 |
| CsbZIP-40 | 5 | AT1G13600.1 | 1 | 4.93 | 0.71 | 0.14 | 37.9 |
| CsbZIP-42 | 5 | AT3G12250.1 | 3 | 1.69 | 0.11 | 0.07 | 13.0 |
| CsbZIP-43 | 5 | AT3G30530.1 | 3 | 4.65 | 0.28 | 0.06 | 35.8 |
| CsbZIP-44 | 6 | AT3G56850.1 | 3 | 4.38 | 0.33 | 0.08 | 33.7 |
| CsbZIP-47 | 6 | AT2G40620.1 | 2 | 4.17 | 0.31 | 0.07 | 32.0 |
| CsbZIP-48 | 6 | AT1G08320.2 | 1 | 3.09 | 0.31 | 0.10 | 23.8 |
| CsbZIP-50 | 6 | AT3G12250.1 | 3 | 1.55 | 0.11 | 0.07 | 11.9 |
| CsbZIP-52 | 6 | AT4G37730.1 | 4 | 3.86 | 0.64 | 0.16 | 29.7 |
| CsbZIP-53 | 6 | AT4G36730.1 | 4 | 2.73 | 0.24 | 0.09 | 21.0 |
| CsbZIP-56 | 7 | AT4G38900.3 | 4 | 3.64 | 0.26 | 0.07 | 28.0 |
| CsbZIP-57 | 7 | AT4G38900.1 | 4 | 3.85 | 0.27 | 0.07 | 29.6 |
| CsbZIP-58 | 7 | AT2G42380.1 | 2 | 2.96 | 0.29 | 0.10 | 22.8 |
| CsbZIP-59 | 7 | AT2G16770.1 | 2 | 3.32 | 0.22 | 0.07 | 25.5 |
| CsbZIP-60 | 7 | AT4G35040.1 | 4 | 5.45 | 0.23 | 0.04 | 41.9 |
| CsbZIP-61 | 7 | AT2G40620.1 | 2 | 4.02 | 0.58 | 0.14 | 30.9 |
| CsbZIP-64 | Scaffold000222 | AT1G68640.1 | 1 | 2.94 | 0.27 | 0.09 | 22.6 |
| Mean |  |  |  | 3.41 | 0.34 | 0.10 | 26.2 |
|  |  |  |  |  |  |  |  |
| **Cucumber -Rice** | | | | | | | |
| **ID** | **Chromosome** | **Gene IDs** | **Chromosome** | **Ks** | **Ka** | **Ka/Ks** | **Mya** |
| CsbZIP-02 | 1 | Os05g37170.3 | 5 | 4.30 | 0.38 | 0.09 | 33.1 |
| CsbZIP-03 | 1 | Os03g58250.1 | 3 | 2.73 | 0.47 | 0.17 | 21.0 |
| CsbZIP-04 | 1 | Os01g59760.1 | 1 | 5.40 | 0.49 | 0.09 | 41.6 |
| CsbZIP-05 | 2 | Os03g58250.1 | 3 | 3.33 | 0.54 | 0.16 | 25.6 |
| CsbZIP-11 | 2 | Os02g07840.2 | 2 | 6.15 | 0.56 | 0.09 | 47.3 |
| CsbZIP-12 | 2 | Os02g10860.1 | 2 | 6.19 | 0.17 | 0.03 | 47.6 |
| CsbZIP-14 | 2 | Os01g55150.1 | 1 | 7.03 | 0.41 | 0.06 | 54.0 |
| CsbZIP-15 | 3 | Os12g13170.2 | 12 | 2.36 | 0.32 | 0.14 | 18.2 |
| CsbZIP-17 | 3 | Os09g34060.1 | 9 | 4.71 | 0.34 | 0.07 | 36.2 |
| CsbZIP-18 | 3 | Os09g34060.1 | 9 | 3.26 | 0.31 | 0.09 | 25.1 |
| CsbZIP-20 | 3 | Os12g09250.1 | 12 | 7.57 | 0.52 | 0.07 | 58.3 |
| CsbZIP-25 | 3 | Os05g34050.1 | 5 | 3.03 | 0.49 | 0.16 | 23.3 |
| CsbZIP-26 | 3 | Os01g17260.2 | 1 | 2.20 | 0.12 | 0.05 | 16.9 |
| CsbZIP-29 | 3 | Os12g13170.1 | 12 | 5.53 | 0.33 | 0.06 | 42.5 |
| CsbZIP-33 | 4 | Os04g54474.2 | 4 | 5.85 | 0.37 | 0.06 | 45.0 |
| CsbZIP-37 | 4 | Os01g55150.1 | 1 | 7.08 | 0.38 | 0.05 | 54.4 |
| CsbZIP-39 | 5 | Os07g48180.1 | 7 | 4.59 | 2.05 | 0.45 | 35.3 |
| CsbZIP-42 | 5 | Os01g59350.1 | 1 | 2.93 | 0.12 | 0.04 | 22.5 |
| CsbZIP-44 | 6 | Os01g59760.1 | 1 | 6.98 | 0.42 | 0.06 | 53.7 |
| CsbZIP-46 | 6 | Os11g06170.2 | 11 | 5.24 | 0.34 | 0.06 | 40.3 |
| CsbZIP-47 | 6 | Os07g48180.1 | 7 | 3.80 | 0.44 | 0.11 | 29.2 |
| CsbZIP-50 | 6 | Os07g48820.1 | 7 | 1.99 | 0.12 | 0.06 | 15.3 |
| CsbZIP-57 | 7 | Os07g48180.1 | 7 | 6.18 | 0.50 | 0.08 | 47.6 |
| CsbZIP-58 | 7 | Os01g55150.1 | 1 | 6.20 | 0.39 | 0.06 | 47.7 |
| CsbZIP-59 | 7 | Os06g50310.1 | 6 | 5.18 | 0.37 | 0.07 | 39.8 |
| CsbZIP-60 | 7 | Os06g50310.1 | 6 | 9.05 | 0.31 | 0.03 | 69.6 |
| CsbZIP-61 | 7 | Os12g06520.1 | 12 | 4.66 | 0.42 | 0.09 | 35.9 |
| CsbZIP-64 | Scaffold000222 | Os01g59350.1 | 1 | 3.96 | 0.32 | 0.08 | 30.5 |
| Mean |  |  |  | 4.91 | 0.43 | 0.10 | 37.8 |
|  |  |  |  |  |  |  |  |
| **Cucumber -Poplar** | | | | | | | |
| **ID** | **Chromosome** | **Gene IDs** | **Chromosome** | **Ks** | **Ka** | **Ka/Ks** | **Mya** |
| CsbZIP-02 | 1 | 009G164300.1 | 9 | 1.53 | 0.20 | 0.13 | 11.8 |
| CsbZIP-03 | 1 | 013G040700.1 | 13 | 1.69 | 0.37 | 0.22 | 13.0 |
| CsbZIP-04 | 1 | 001G220700.2 | 1 | 1.12 | 0.23 | 0.21 | 8.6 |
| CsbZIP-05 | 2 | 005G053200.1 | 5 | 1.60 | 0.33 | 0.20 | 12.3 |
| CsbZIP-06 | 2 | 009G119700.1 | 9 | 2.61 | 0.28 | 0.11 | 20.0 |
| CsbZIP-07 | 2 | 014G013400.1 | 14 | 1.83 | 0.36 | 0.20 | 14.1 |
| CsbZIP-08 | 2 | 014G028200.1 | 14 | 1.85 | 0.21 | 0.11 | 14.3 |
| CsbZIP-09 | 2 | 005G231300.1 | 5 | 4.35 | 0.22 | 0.05 | 33.5 |
| CsbZIP-11 | 2 | 006G277800.6 | 6 | 3.36 | 0.41 | 0.12 | 25.8 |
| CsbZIP-12 | 2 | 018G029500.1 | 18 | 1.79 | 0.08 | 0.04 | 13.8 |
| CsbZIP-13 | 2 | 002G090700.1 | 2 | 1.64 | 0.23 | 0.14 | 12.6 |
| CsbZIP-14 | 2 | 019G091900.1 | 19 | 2.60 | 0.33 | 0.13 | 20.0 |
| CsbZIP-15 | 3 | 001G136000.1 | 1 | 0.92 | 0.12 | 0.14 | 7.0 |
| CsbZIP-17 | 3 | 007G130800.1 | 7 | 2.00 | 0.18 | 0.09 | 15.3 |
| CsbZIP-18 | 3 | 017G027400.1 | 17 | 1.78 | 0.17 | 0.09 | 13.7 |
| CsbZIP-19 | 3 | 003G014800.1 | 3 | 1.31 | 0.19 | 0.14 | 10.0 |
| CsbZIP-20 | 3 | 003G014800.1 | 3 | 1.78 | 0.25 | 0.14 | 13.7 |
| CsbZIP-22 | 3 | 006G083000.1 | 6 | 2.55 | 0.25 | 0.10 | 19.6 |
| CsbZIP-23 | 3 | 006G114600.1 | 6 | 1.69 | 0.47 | 0.28 | 13.0 |
| CsbZIP-24 | 3 | 006G058800.1 | 6 | 1.58 | 0.09 | 0.06 | 12.1 |
| CsbZIP-25 | 3 | 016G032400.1 | 16 | 1.95 | 0.37 | 0.19 | 15.0 |
| CsbZIP-26 | 3 | 016G036500.1 | 16 | 1.06 | 0.12 | 0.12 | 8.1 |
| CsbZIP-27 | 3 | 005G231300.1 | 5 | 2.76 | 0.28 | 0.10 | 21.2 |
| CsbZIP-28 | 3 | 006G251800.2 | 6 | 2.02 | 0.24 | 0.12 | 15.6 |
| CsbZIP-29 | 3 | 003G097600.1 | 3 | 1.28 | 0.19 | 0.15 | 9.9 |
| CsbZIP-32 | 4 | 004G158200.1 | 4 | 2.20 | 0.22 | 0.10 | 17.0 |
| CsbZIP-33 | 4 | 007G085700.1 | 7 | 1.60 | 0.20 | 0.12 | 12.3 |
| CsbZIP-37 | 4 | 001G374200.1 | 1 | 1.81 | 0.35 | 0.19 | 13.9 |
| CsbZIP-38 | 4 | 014G013400.1 | 14 | 2.10 | 0.35 | 0.17 | 16.2 |
| CsbZIP-39 | 5 | 013G156900.3 | 13 | 3.43 | 1.62 | 0.47 | 26.4 |
| CsbZIP-40 | 5 | 010G135200.1 | 10 | 2.47 | 0.45 | 0.18 | 19.0 |
| CsbZIP-42 | 5 | 003G194600.1 | 3 | 1.11 | 0.06 | 0.06 | 8.5 |
| CsbZIP-43 | 5 | 004G111100.1 | 4 | 1.68 | 0.23 | 0.14 | 12.9 |
| CsbZIP-44 | 6 | 008G010800.1 | 8 | 1.35 | 0.14 | 0.10 | 10.4 |
| CsbZIP-46 | 6 | 002G069500.1 | 2 | 2.3748 | 0.2614 | 0.11 | 18.3 |
| CsbZIP-47 | 6 | 013G156900.1 | 13 | 1.95 | 0.20 | 0.10 | 15.0 |
| CsbZIP-48 | 6 | 009G164300.1 | 9 | 1.98 | 0.32 | 0.16 | 15.2 |
| CsbZIP-49 | 6 | 009G164500.1 | 9 | 2.71 | 0.41 | 0.15 | 20.9 |
| CsbZIP-50 | 6 | 001G029800.1 | 1 | 1.08 | 0.06 | 0.06 | 8.3 |
| CsbZIP-53 | 6 | 007G029400.1 | 7 | 1.50 | 0.22 | 0.15 | 11.5 |
| CsbZIP-55 | 7 | 004G140600.1 | 4 | 1.48 | 0.31 | 0.21 | 11.4 |
| CsbZIP-56 | 7 | 004G163800.1 | 4 | 1.82 | 0.18 | 0.10 | 14.0 |
| CsbZIP-57 | 7 | 004G163800.1 | 4 | 1.99 | 0.18 | 0.09 | 15.3 |
| CsbZIP-58 | 7 | 013G124400.1 | 13 | 2.10 | 0.26 | 0.12 | 16.1 |
| CsbZIP-59 | 7 | 009G134900.1 | 9 | 2.28 | 0.23 | 0.10 | 17.5 |
| CsbZIP-60 | 7 | 009G134900.1 | 9 | 2.88 | 0.18 | 0.06 | 22.1 |
| CsbZIP-61 | 7 | 002G069500.1 | 2 | 1.98 | 0.26 | 0.13 | 15.2 |
| CsbZIP-62 | 7 | 005G053200.1 | 5 | 4.26 | 0.59 | 0.14 | 32.8 |
| CsbZIP-64 | Scaffold000222 | 008G118300.1 | 8 | 1.87 | 0.19 | 0.10 | 14.4 |
| Mean | | | | 2.01 | 0.28 | 0.14 | 15.5 |
