# Supplementary material for: Genome-Wide Analysis of the bZIP Transcription Factors in Cucumber
Source: PLoS One. 2014 Apr 23;9(4):e96014. doi: 10.1371/journal.pone.0096014 (PMC3997510; doi:10.1371/journal.pone.0096014)
Supplement: Table S8 — List of primers used in quantitative real-time-PCR expression analysis of bZIP genes. (DOC) [file pone.0096014.s011.doc]

**Table S8.** List of primers used in quantitative real-time-PCR expression analysis of bZIP genes.

| **Name** | **Forward Primers (5′- 3′)** | **Reverse Primers (5′- 3′)** |
| --- | --- | --- |
| CsbZIP-06 | GCCCGGAGATCCAGAATGAG | GTGTGGTGACGGCGAGATTA |
| CsbZIP-08 | GAACCAAGAACTCCGGCAGA | GCCAGTCTGCGTTCGTCTTA |
| CsbZIP-12 | TTCTCGGGAGGGTCAAAGGA | TCTCTCCCTTGCTTGCTGTG |
| CsbZIP-15 | GCTGTCATCCCATTGGCAAC | CGCCCCAATAGTCCATTCCA |
| CsbZIP-29 | TGAAGGCACGAGTGAACGAA | CCATTAGGAGTCCCTCCACC |
| CsbZIP-30 | GTCTGCGCTCCTTGAACTCT | GAACTGGGCGAGAGAACTCC |
| CsbZIP-44 | GACCGTTGATGAGGTGTGGA | CACCCCTGCTTTCACCAGAA |
| CsbZIP-53 | CTCCAACTCCGCACCCTTAC | GGATGGGCATAAACTCCCCC |
| CsbZIP-55 | CCCCATTTCGTGATTCCCCA | ACTGGCATCCCTATCCTCCA |
| CsbZIP-59 | ATCAGCAAGACGCTCCAGAC | TCAACAGCAGCATCGTCGTA |
